# Comparison of Indoor Mercury Vapor in Common Areas of Residential Buildings with Outdoor Levels in a Community Where Mercury Is Used for Cultural Purposes

**DOI:** 10.1289/ehp.8410

**Published:** 2005-09-20

**Authors:** Gary Garetano, Michael Gochfeld, Alan H. Stern

**Affiliations:** 1 Hudson Regional Health Commission, Secaucus, New Jersey, USA; 2 Department of Environmental and Occupational Health, University of Medicine and Dentistry of New Jersey–School of Public Health, Piscataway, New Jersey, USA; 3 Department of Environmental and Occupational Medicine, University of Medicine and Dentistry of New Jersey–Robert Wood Johnson Medical School, Piscataway, New Jersey, USA; 4 Environmental and Occupational Health Sciences Institute, Piscataway, New Jersey, USA; 5 New Jersey Department of Environmental Protection, Division of Science, Research, and Technology, Trenton, New Jersey, USA

**Keywords:** cultural use of mercury, elemental mercury, indoor air quality, mercury, mercury exposure, mercury vapor, Santeria, voodoo

## Abstract

Elemental mercury has been imbued with magical properties for millennia, and various cultures use elemental mercury in a variety of superstitious and cultural practices, raising health concerns for users and residents in buildings where it is used. As a first step in assessing this phenomenon, we compared mercury vapor concentration in common areas of residential buildings versus outdoor air, in two New Jersey cities where mercury is available and is used in cultural practices. We measured mercury using a portable atomic absorption spectrometer capable of quantitative measurement from 2 ng/m^3^ mercury vapor. We evaluated the interior hallways in 34 multifamily buildings and the vestibule in an additional 33 buildings. Outdoor mercury vapor averaged 5 ng/m^3^; indoor mercury was significantly higher (mean 25 ng/m^3^; *p* < 0.001); 21% of buildings had mean mercury vapor concentration in hallways that exceeded the 95th percentile of outdoor mercury vapor concentration (17 ng/m^3^), whereas 35% of buildings had a maximum mercury vapor concentration that exceeded the 95th percentile of outdoor mercury concentration. The highest indoor average mercury vapor concentration was 299 ng/m^3^, and the maximum point concentration was 2,022 ng/m^3^. In some instances, we were able to locate the source, but we could not specifically attribute the elevated levels of mercury vapor to cultural use or other specific mercury releases. However, these findings provide sufficient evidence of indoor mercury source(s) to warrant further investigation.

Mercury is one of two elements that are liquid at ambient temperature. It is 13 times heavier than water, and its unique properties have led to a wide variety of uses in industry and elsewhere. Elemental mercury is still widely used in dentistry and a variety of hospital applications (Haas et al. 2003). It is also found in a number of technologic applications such as thermometers, barometers, thermostats, switches, gas meters, and especially fluorescent lights that may be found in residential buildings. In the past, organic mercury compounds were widely used as preservatives in household paints, and mercury antiseptics are still in use.

The unique properties of elemental mercury or quicksilver have led people to attribute magical and spiritual powers to it through the ages. Mercury was viewed as an essential component of the alchemical triad of mercury, sulfur, and air and has been associated with the Hindu god Shiva (Little 1997). Mercury amalgam religious icons remain available today (Garetano G, unpublished data). Elemental mercury is also used in the spiritual practices associated with Santeria, voodoo, Espiritismo, Palo Mayumbo, and other Afro-Caribbean syncretic religions [Riley et al. 2001; U.S. Environmental Protection Agency (EPA) 2002]. Additional uses of elemental mercury in a superstitious manner have been reported (Wendroff 1990). These practices include sprinkling elemental mercury in the home, in cars, or around babies and carrying capsules of mercury as amulets to bring good luck or love (Johnson 1999; U.S. EPA 2002). These activities do not appear to be components of ceremonial use associated with spiritual traditions, nor are they condoned or recommended by serious practitioners of those traditions (Stern et al. 2003). We label these uses of mercury, separate from the ceremonial use in spiritual traditions, as cultural uses. In communities where cultural uses of mercury are believed to be prevalent, the availability of mercury in specialty shops called botanicas has been well documented (Riley et al. 2001; Wendroff 1990; Zayas and Ozuah 1996).

Both the technologic applications and cultural uses of mercury provide the opportunity for it to be an indoor air pollutant in residential settings. Elemental mercury evaporates at a rate of 7 μg/cm^2^/hr at 20°C (Andren and Nriagu 1979). Up to 80% of inhaled mercury is absorbed and readily crosses the blood–brain barrier (Cherian et al. 1978; Clarkson 2002). The primary health concern associated with inhaled mercury vapor is its neurotoxicity, and infants are considered particularly vulnerable. The Agency for Toxic Substances and Disease Registry (ATSDR) and the U.S. EPA, respectively, have established a minimal risk level (MRL) of 300 ng/m^3^ and a reference concentration (RfC) of 200 ng/m^3^ for elemental mercury vapor in residential quarters (ATSDR 1999; U.S. EPA 1995). The release of elemental mercury in a household may pose some health risk for those who are exposed. For example, broken clinical thermometers typically contain only 600–675 mg elemental mercury but can generate mercury vapor concentrations an order of magnitude above both the U.S. EPA RfC and the ATSDR MRL (Carpi and Chen 2001; Muhlendahl 1990; Riley et al. 2001; Smart 1986). Health effects in children have been documented from such exposures (Moreno-Ramírez et al. 2004).

By comparison, elemental mercury for cultural use is commonly distributed in gelatin capsules containing approximately 9 g elemental mercury (Riley et al. 2001; Wendroff 1990), which, when released, can result in high concentrations of vapor (Riley et al. 2001; U.S. EPA 1993). At least one case of significant human exposure to elemental mercury requiring medical intervention as a result of cultural practices has been reported (Forman et al. 2000).

Once spilled, sprinkled, or left in an open container, elemental mercury may release vapor for prolonged periods. Significant levels of mercury vapor have been found in buildings decades after spillage, resulting in the significant exposure of subsequent building occupants without their knowledge (Centers for Disease Control and Prevention 1996; Orloff et al. 1997).

Other than those investigations conducted in response to known spills, data regarding mercury vapor concentration in residential buildings are scant. Carpi and Chen (2001) surveyed 12 residential and commercial sites in the New York metropolitan area without prior knowledge of mercury contamination. Eleven of these locations were found to have mercury vapor concentrations significantly elevated over outdoor concentrations. Prior breakage of clinical fever thermometers was subsequently identified as the probable mercury source in two of the locations.

Given the lack of documentation of mercury vapor in residential buildings in general or of a disproportionate elevation of mercury vapor in buildings in communities where it is used culturally, we chose to conduct a survey of residential dwellings in a community in which elemental mercury is readily available to assess the prevalence of mercury use or spillage.

We hypothesized that elevated levels of mercury vapor would be found in residential buildings in communities that engage in cultural uses of mercury. We further hypothesized that these elevated levels can serve as a signal of significant cultural use in addition to unintentional breakage and spillage from other sources. In this article we address the first hypothesis. We address the second hypothesis in a subsequent study to be published separately.

## Materials and Methods

### Rationale for this study design.

Riley et al. (2001) described a high level of apprehension and distrust of authorities or any outsider from a different culture. As a result of these cultural barriers, direct investigation of the residences of persons possibly using mercury for cultural purposes without first establishing a cause for concern was deemed inappropriate. Therefore, as a first step in characterizing the extent of this phenomenon, we chose to monitor mercury vapor within interior hallways of residential buildings, rather than directly measuring mercury vapor in residences, under the assumption that intentional and unintentional releases of mercury within the building would be reflected in elevated concentrations in common areas compared with the respective outdoor concentrations. Measurement of mercury vapor in common areas does not provide a direct estimate of exposure, but by comparing these measurements with respective outdoor levels and by comparing measurements across buildings, we can assess the prevalence of elevated indoor mercury concentrations. This information can inform decisions about appropriate public health strategies and can guide future surveys.

### Site selection.

The information on cultural uses of mercury suggests that such uses are most common among certain Latino-Caribbean populations. The geographic area selected for inquiry was based on our prior knowledge of both the predominant Latino population and the presence of botanicas that typically sell mercury (Riley et al. 2001; Stern et al. 2003). The study was conducted in the New Jersey municipalities of Union City and West New York, comprising a total area of approximately 2.4 mi^2^ (6.2 km^2^), with 82.3 and 78.7% Latino population, respectively. Multifamily buildings were chosen for accessibility of common areas as well as for the potential for efficient screening. A primary criterion was that the buildings surveyed be within 0.5 miles (0.8 km) of a botanica. On the initial sampling date, a building meeting this criterion was selected on referral from a local health official, and all accessible buildings for approximately a two-block radius were evaluated. On subsequent sampling dates the same procedure was followed in other areas of the community meeting the same criteria. Additionally, three botanicas and one former botanica encountered during the residential building surveys were also visited.

### Mercury vapor monitoring.

We measured real-time mercury vapor concentration in air using an atomic absorption spectrometer (model 915+; Ohio Lumex Co. Inc., Twinsburg, OH). The instrument has a sensitivity of 2 ng/m^3^ of mercury in air and has been successfully used for measuring mercury in ambient air (Ohio Lumex 2000; Zdravko and Mashyanov 2000). In previous studies, residential structures identified as having elevated mercury concentration with such direct reading instruments were also found to have elevated mercury vapor concentration with 8-hr sampling and subsequent laboratory analysis (Singhvi et al. 2001).

The instrument was factory calibrated according to the manufacturer’s specification and was within its factory calibration schedule. The spectrometer warmup, operation, and calibration followed the manufacturer’s instructions. Internal calibration uses a builtin mercury cell and was performed in the field before and on completion of sampling in typical field conditions. During internal calibration, measured mercury concentration varied from the predicted concentration by < 10% on each date. We validated precision by evaluating the relative deviation of triplicate measurements at each sampling location. The overall relative deviation for the 286 triplicate sample sets that were equal to or exceeding the manufacturers’ stated detection limit of 2 ng/m^3^ mercury vapor was 7.9%. Once the instrument was warmed up and calibrated, it was operated continuously. All measurements were recorded at a height of approximately 1 m above the floor unless otherwise indicated. Each data point is the average of three discrete 10-sec measurements at a given sampling location. The instrument also displayed mercury concentration continuously in a real-time sampling mode. This allowed evaluation of spatial variation and trends in mercury vapor concentration. Potential sources were localized where possible.

Site visits were conducted on 6 days in June and August 2002. Although only one visit was planned for each site, repeat visits were made to two buildings because of the high mercury vapor concentration encountered. Mercury vapor was monitored in the vestibule and the interior hallways on each floor of the buildings. These interior hallways contain the entrances to residential apartments. About half the buildings surveyed had open access to both locations. A total of 227 locations in 67 buildings were surveyed. On average, five hallway locations were assessed in those buildings that were fully accessible. All buildings were visited once except the two buildings with the highest readings. Mercury vapor measurements were recorded in 37 outdoor locations in proximity to the buildings evaluated. Outdoor readings near neighboring buildings showed low variation. Within the three botanicas and one former botanica, mercury vapor was monitored in the retail portion of the store.

### Additional data.

In addition to mercury vapor measurements, the following data were also collected for each building: number of residential units, number of floors, presence of a central heating ventilation and air conditioning system (HVAC), and the presence of open windows.

### Data analysis.

We calculated the mean mercury vapor concentration for each floor of a building by averaging all data points for that floor. We computed the average mercury concentration for a building by averaging the mean concentration for each floor. The maximum mercury vapor concentration reported for a building is the maximum data point from any hallway location within the building. Statistical analysis was conducted using SPSS software (SPSS Inc., Chicago, IL). Specific tests are indicated in the results section as applicable.

## Results

### Site access and characteristics.

Sixty-seven buildings were visited, of which approximately half were fully accessible. Only vestibules were accessible in the remainder. All buildings in which the interior halls were was accessed (*n* = 34) were multistory (mean, 4 floors) with a total of 497 residential units (mean, 14 units). Buildings in which only the vestibule was accessible tended to be slightly smaller (mean, 12 units), although this difference was not significant (*p* = 0.18). Based on familiarity with the area, including community history, overall appearance, and census characteristics, all buildings are believed to be > 50 years old, although records were not uniformly available. None of the buildings had HVAC systems that influenced the areas evaluated. Ventilation within the hallways was primarily influenced by windows and doors to residential apartments; 12 of 34 (35%) buildings had open hallway windows during the time of the visit.

### Mercury vapor concentration.

The data were log-normally distributed; thus, arithmetic and geometric mean values, as well as percentiles, are reported. Because of relatively limited sample size and non-normal distributions, we compared mercury values using the Mann-Whitney *U*-test as well as by *t*-test on log-transformed data, unless otherwise indicated.

Outdoor mercury vapor concentrations had a mean value of 5 ng/m^3^ with an 80th percentile of 12 ng/m^3^ and a 95th percentile of 17 ng/m^3^. Our findings are consistent with outdoor levels measured elsewhere ranging from several nanograms per cubic meter to 20 ng/m^3^, with higher concentrations associated with urban/industrial areas and ambient mercury outside a mercury storage facility in Hillsborough, New Jersey, ranging from 2 to 8 ng/m^3^ (ATSDR 1999; Gochfeld M, unpublished data; New Jersey Department of Environmental Protection 2001).

The geometric and arithmetic mean mercury concentrations in building hallways were 10 ng/m^3^ and 25 ng/m^3^, respectively. In building vestibules, the geometric and arithmetic means were 7 ng/m^3^ and 11 ng/m^3^, respectively. The mercury vapor concentration in interior hallways was significantly greater than that found outdoors (*p* < 0.001) and in building vestibules (*p* < 0.05). Mercury vapor in vestibules was also greater than that found outdoors (*p* < 0.001). All three locations were found to differ significantly (*p* < 0.001) when compared simultaneously using the Kruskal-Wallis nonparametric one-way analysis of variance test. Indoor and outdoor mercury vapor concentrations are summarized in Tables 1 and 2.

### Spatial variability.

We were able to localize potential sources of mercury contamination in seven buildings as evidenced by increasing mercury concentration as the “source area” was approached. At two sites, the probable source of mercury vapor emission was tracked to areas on the floor surface, one near a building entrance, the second on a stairway to a roof exit. In the remaining five buildings, mercury vapor concentration increased as certain individual or groups of apartment entrances were approached. No visible contamination was noted in any of the cases, and the actual source of vapor remained unknown.

We noted order of magnitude differences in mercury concentration between locations in buildings with high mercury concentration. For example, mercury vapor concentration ranged from 35 ng/m^3^ to 2,022 ng/m^3^ in the building with the highest concentration. Similar findings were noted elsewhere. The difference between mercury concentration on the building level (floor) on which the maximal value was noted and the remainder of the building was significantly higher in four of the buildings (*p* < 0.04).

### Temporal variability.

Although our intent was to survey buildings once, two buildings had maximum hallway mercury vapor concentrations of 2,022 ng/m^3^ and 774 ng/m^3^, which exceeded both the ATSDR MRL (300 ng/m^3^) and U.S. EPA RfC (200 ng/m^3^). Local public health officials were notified, and repeat visits were made to each building. The building with the highest concentration was visited on five dates. Both the average and maximum mercury vapor concentrations of the building were significantly different on repeat visits (Kruskal-Wallis test, *p* < 0.04). Outdoor temperature ranged from 17 to 31°C, and hallway windows were open, providing passive ventilation, on all dates. The building hallways were not cooled, and indoor temperature was similar to that outdoors. Unexpectedly, mercury vapor concentration did not vary as a result of temperature changes (*p* > 0.7), and contrary to expectation, higher mercury vapor concentrations were noted on cooler days. By the final visit, maximum mercury vapor concentrations in each building (109 and 19 ng/m^3^, respectively) were significantly reduced (*p* < 0.01) compared with the initial visit. In both buildings, mean and maximum mercury concentrations fell below MRL and RfC. Despite the reduction in vapor concentration, the area of maximum concentration remained consistent.

## Discussion

Our findings provide a valuable first look at the differences between indoor mercury concentrations and those outdoors in an area with known cultural use of mercury. Although our data are not intended as estimates of residential exposure to mercury vapor, they do indicate that, compared with outdoor levels, such exposures are likely in a significant proportion of multifamily residential buildings in an area with known cultural uses of mercury. This study did not include comparison with indoor mercury concentrations in a comparable area that can serve as a control for cultural use of mercury. Therefore, these data cannot distinguish between those elevations in mercury concentration resulting from cultural uses and those resulting from unintentional releases of mercury (e.g., broken thermometers or fluorescent lightbulbs, spilled gas meter seals). We are currently engaged in a follow-up study to investigate these questions.

There are relatively few reports of “background” mercury concentration in indoor air in residential buildings or “noncontaminated” environments to which our results can be compared. Our finding of mercury vapor in greater concentrations indoors compared with outdoors is consistent with the findings of Carpi and Chen (2001), who investigated mercury in residences without prior knowledge of mercury use or release.

Carpi and Chen (2001), using a direct reading instrument, were able to identify specific points inside several of the apartments they investigated that appeared to be the source of mercury emissions. Likewise, we were able to localize potential mercury sources in several buildings with elevated mercury concentrations. We clearly observed an increasing gradient in mercury vapor concentration as a potential source was approached. Although the exact source was not identified, the potential source of mercury vapor seemed to be residential apartments in five of the buildings with elevated mercury vapor concentration. Our finding that > 20% of buildings we studied had average and 35% had maximum mercury vapor concentrations that exceed the 95th percentile of outdoor concentrations is significant and leads to the conclusion that sources of contamination are present and prevalent indoors in this community. These findings are consistent with the hypothesis of cultural use of mercury, but not definitive. The elevated mercury vapor concentration found in botanicas is also consistent with its availability for cultural use.

These measurements were not made in areas that directly reflect exposure, nor, for the most part, do they measure concentration at the emission source. Therefore, these measurements could underestimate mercury concentration at the point of long-term exposure. Our surveys were subject to the variability in environmental conditions that occurs in occupied residential buildings and possibly the variability in patterns and methods of cultural mercury use. In most buildings surveyed, including those with the highest mercury vapor concentration, windows were open. This may partially explain the variability in mercury concentration and the lack of association with temperature we found in the sites with repeated visits. Although spot measurements of mercury vapor concentration in buildings may not reflect long-term average mercury concentration, we believe that the signals of elevated mercury concentration provided by spot measurements are relevant as a screening tool in identifying the presence of mercury release regardless of its source. For this approach to be more effective as a tool for screening for exposures of concern, models need to be developed that can reasonably predict the transit of mercury vapor from a source “behind closed doors” to other rooms or areas of a building under conditions that simulate occupancy.

Whether exposure to elevated mercury vapor arises from intentional cultural uses or from unintentional breakage and spillage of mercury-containing equipment, these exposures pose the potential for adverse health effects and should be addressed. However, the nature and scope of the public health problem will be significantly different for each of these cases. Each will require a different public health outreach and intervention strategy. It is therefore essential that future investigations clarify the relative contribution of each cause. We are currently continuing research to this end.

Given the findings of Carpi and Chen (2001) and this investigation, we feel some broader evaluations to establish reference ranges of mercury concentrations in the indoor residential environment are warranted. Such a reference range would include mercury contamination resulting from historical accidental breakage of mercury-containing equipment. Such contamination may be widespread and would likely be independent of cultural factors. Based on reports on the manner in which mercury may be used for cultural purposes, and our present findings, we also recommend expanded screenings in areas where mercury may be used for cultural purposes with the inclusion of suitable control locations. Although cultural obstacles may be present that may impede a direct approach to assessing human exposure to mercury vapor as a result of cultural practices and its relevance to public health, we believe further evaluations in the field will ultimately shed light on this elusive issue.

## Figures and Tables

**Table 1:** Comparison of mercury vapor concentration (ng/m^3^) within building hallways and outdoors.

Location	No.	Arithmetic mean ± SD	Geometric mean (SD)
Outdoors	37	5 ± 5	4 (2)
Building vestibule	57	11 ± 12	7 (2)
Mean in building hallways	34	25 ± 53	10 (4)
Maximum in building hallways	34	102 ± 364	17 (4)

Mann-Whitney *U*-test, *p* < 0.001.

**Table 2:** Distribution of mercury vapor concentration (ng/m^3^) within building hallways and outdoors.

	Percentile				
Location	25th	50th	75th	90th	95th
Outdoors	3	4	6	12	17
Building vestibules	4	7	13	22	36
Mean of building hallways	6	11	16	66	155
Maximum within hallways	9	14	25	106	1,086
